# Developmental differences in amygdala projection neuron activation associated with isolation-driven changes in social preference

**DOI:** 10.3389/fnbeh.2022.956102

**Published:** 2022-08-24

**Authors:** Nicole C. Ferrara, Sydney Trask, Alexandra Ritger, Mallika Padival, J. Amiel Rosenkranz

**Affiliations:** ^1^Department of Foundational Sciences and Humanities, Discipline of Cellular and Molecular Pharmacology, Chicago Medical School, Rosalind Franklin University of Medicine and Science, North Chicago, IL, United States; ^2^Center for Neurobiology of Stress Resilience and Psychiatric Disorders, Rosalind Franklin University of Medicine and Science, North Chicago, IL, United States; ^3^Department of Psychological Sciences, Purdue University, West Lafayette, IN, United States

**Keywords:** amygdala, isolation, adolescent development, social interaction, social preference

## Abstract

Adolescence is a developmental period characterized by brain maturation and changes in social engagement. Changes in the social environment influence social behaviors. Memories of social events, including remembering familiar individuals, require social engagement during encoding. Therefore, existing differences in adult and adolescent social repertoires and environmentally-driven changes in social behavior may impact novel partner preference, associated with social recognition. Several amygdala subregions are sensitive to the social environment and can influence social behavior, which is crucial for novelty preference. Amygdala neurons project to the septum and nucleus accumbens (NAc), which are linked to social engagement. Here, we investigated how the social environment impacts age-specific social behaviors during social encoding and its subsequent impact on partner preference. We then examined changes in amygdala-septal and -NAc circuits that accompany these changes. Brief isolation can drive social behavior in both adults and adolescents and was used to increase social engagement during encoding. We found that brief isolation facilitates social interaction in adolescents and adults, and analysis across time revealed that partner discrimination was intact in all groups, but there was a shift in preference within isolated and non-isolated groups. We found that this same isolation preferentially increases basal amygdala (BA) activity relative to other amygdala subregions in adults, but activity among amygdala subregions was similar in adolescents, even when considering conditions (no isolation, isolation). Further, we identify isolation-driven increases in BA-NAc and BA-septal circuits in both adults and adolescents. Together, these results provide evidence for changes in neuronal populations within amygdala subregions and their projections that are sensitive to the social environment that may influence the pattern of social interaction within briefly isolated groups during development.

## Introduction

Younger populations require socially enriching experiences, and these experiences have a lasting impact on a variety of behaviors, namely, cognitive flexibility, anxiety-like behaviors, and social responses (Hol et al., 1999; Hellemans et al., 2004; Varlinskaya and Spear, 2008; Amitai et al., 2014; Butler et al., 2016; Schneider et al., 2016). Adolescence, in particular, is a key period of time with heightened social sensitivity across several species (Varlinskaya and Spear, 2008; Seffer et al., 2015; Foulkes and Blakemore, 2016; Orben et al., 2020; Ferrara et al., 2021a). Alterations in social behavior characterize several neuropsychiatric disorders, with depression and anxiety often characterized by social avoidance and withdrawal (Rankin et al., 2008; Trew, 2011; Meyer-Lindenberg and Tost, 2012; Fernández-Theoduloz et al., 2019). Diagnosis rates are high during adolescence suggesting susceptibility, and there are added negative outcomes of reduced social engagement on long-term behavioral responses in those diagnosed (Gerhard et al., 2021). The social environment influences social function and can bidirectionally influence neuropsychiatric disorder diagnosis (Meyer-Lindenberg and Tost, 2012). A clear understanding of the behavioral and biological mechanisms sensitive to the social environment may inform neuropsychiatric and developmental disorders characterized by changes in social function.

Many behaviors change over the course of development (Campbell and Spear, 1972; Spear, 2000; Douglas et al., 2004; Pattwell et al., 2012; Loh et al., 2022). Adolescence is characterized by transitions in social engagement, where social play is gradually replaced by increases in social investigation [e.g., nose–body and anogenital sniffing; Hol et al. (1999); Varlinskaya and Spear (2008), and Ferrara et al. (2021b)]. Disruptions to the social repertoire especially during social maturation can have detrimental effects on socialization (Hol et al., 1999; Schneider et al., 2016). Manipulations of the social environment through deprivation or isolation can have a bidirectional impact on behavior. Long-term social disruption can impair social learning and interaction, and shorter-term isolation can facilitate age-specific social behaviors during adolescence (Ferrara et al., 2021b; Kinley et al., 2021; Lee et al., 2021; Caruso et al., 2022). This facilitation of social behavior with brief isolation can improve social recognition memory in adolescents, while reducing it in adults, which is indexed with increased novel partner investigation relative to a familiar (Ferrara et al., 2022). This increase in novel partner investigation relative to a familiar is otherwise known as novel partner preference and requires recognition memory and the ability to discriminate between a novel and familiar partner. The impact of brief isolation on social recognition memory tasks might point to an additional influence of social partner preferences that are sensitive to heightened social states. However, the behavioral mechanisms related to social recognition and the impact of isolation are unclear, as isolation-driven facilitation of social interaction may promote shifts in partner preference unrelated to social recognition. This same isolation can engage a network of developing brain regions and may, therefore, influence partner preference from adolescence to adulthood (Lee et al., 2021).

The amygdala is sensitive to and regulates social behavior (Twining et al., 2017; Hu et al., 2021; Opendak et al., 2021; Terranova et al., 2022). Brief isolation can increase neuronal and microglial activity within both lateral (LA) and basal (BA) regions of the amygdala (Ferrara et al., 2021b,^2022^). The medial and basomedial amygdala subregions (MeA and BMA, respectively) are sensitive to the social environment and are critical for social memory, and may, therefore, be similarly sensitive to brief isolation (Mesquita et al., 2016; Twining et al., 2017). However, the amygdala circuits sensitive to the isolation that affect social memory over the course of development are unclear. Neurons from the amygdala project to many regions, including the septum and the nucleus accumbens (NAc), and these brain regions regulate several social behaviors critical for social memory, ranging from social preference to reward (Caffe et al., 1987; McDonald, 1991; Calderazzo et al., 1996; Trezza et al., 2011; Dölen et al., 2013; Kohls et al., 2013; Lukas et al., 2013; Bredewold et al., 2015; Clemens et al., 2020; Hintiryan et al., 2021). Locally, both the septum and NAc are sensitive to social stressors, and their activity can influence social memory processes (Hodges et al., 2017, 2019; Kopec et al., 2018; Folkes et al., 2020). The amygdala, septum, and NAc have been linked to age-specific social behaviors that change from adolescence to adulthood, including social play (Trezza et al., 2012; Kopec et al., 2018; Clemens et al., 2020; Ferrara et al., 2021b), while amygdala inputs to the septum and NAc can regulate social memory and sociability, respectively (Willuhn et al., 2014; Rodriguez et al., 2021). Adolescents are more sensitive to shifted social environments. In addition, a simple shift in the social environment, brief social isolation, is linked to less novel partner preference in social recognition memory tasks in adults and more preference in adolescents. Therefore, amygdala-septum and amygdala-NAc pathways may be more sensitive to isolation in adolescents and influence social memory differentially in adults and adolescents.

In the following experiments, we tested the social behaviors impacted by isolation in the context of novel partner preference within isolated and non-isolated adults and adolescents. We then used a retrobead approach combined with immediate early gene expression to quantify changes in the pattern of overall activity in amygdala subregions and the activity of neurons projecting to the NAc and septum in isolated adults and adolescents. These results identify patterns within, but not between, isolated and non-isolated cohorts of social interaction and degree of partner preference, and the amygdala circuits sensitive to this environmental shift.

## Materials and methods

Experiments were approved by the Institutional Animal Care and Use Committee at Rosalind Franklin University of Medicine and Science and abided by the National Research Council (US) Committee for the Update of the Guide for the Care and Use of Laboratory Animals (2011).

### Subjects

Subjects were male Sprague Dawley rats purchased from Envigo (adolescent *n* = 49, adult *n* = 48; Indianapolis, IN) and housed 2–3 per cage in the Rosalind Franklin University animal facility. Rats had free access to food and water at all times and were maintained on a reverse light cycle (12 h light/dark). Adolescent rats arrived at the animal facility on postnatal days (PND) 20–21, and adult rats arrived on PND 64–69. Adolescents were between the ages of PND 28–38, and adult rats were PND > 70 for behavioral and immunofluorescent experiments.

### Partner preference training, testing, and scoring

Open field behavior and social interaction were measured in a dimly lit room (10–15 lux white light and dim red light). Transparent Plexiglas cages (dimensions 28.58 cm × 17.78 cm × 20.32 cm) used for brief isolation contained bedding and were at least 12 inches from all other cages in the animal colony [as seen in Ferrara et al. (2021b)]. Rats were randomly assigned to 2-h isolation or control conditions, where isolated groups were placed into Plexiglas cages for 2 h, and control groups were left in the home cage. All rats were then acclimated to a black opaque plexiglass open field apparatus for 5 min immediately before social interaction, used here as familiarization for the partner preference task. During this familiarization period, a novel age-matched same-sex conspecific partner was placed into the apparatus, and rats were allowed to freely interact for 5 min. Rats were then brought back to their home cages. After 30 min, rats were returned to the open field and were allowed to investigate the previous stimulus rat (familiar) and a new age-matched same-sex rat (novel) under separate wire cages on the opposite sides of the open field apparatus for 5 min.

All interactions were video recorded and captured with ANY-maze software (Stoelting, Wood Dale, IL, United States). Videos were then uploaded into CowLog software (3.0.2; Hänninen and Pastell, 2009) and scored for social interaction consisting of nose–body contact, anogenital sniffing, play, and chase behaviors during social interaction (the familiarization component of partner preference test). During the partner preference test, the social investigation was scored as the nose touching the cage of a novel or familiar partner. All scoring was by a rater blind to condition [as in Ferrara et al. (2022)]. Data were exported in a CSV file and then transferred to an Excel file (Microsoft, Redmond, WA, United States) where the sum of each interaction was calculated.

Discrimination index was calculated as the amount of time investigating a novel partner subtracted from a familiar partner, and this difference was then divided by the total time spent investigating both partners, then multiplied by 100. Cumulative time spent interacting was calculated for each individual animal by summing the time spent investigating a novel *or* familiar partner for each minute and dividing it by the total time spent investigating a novel *or* familiar partner, respectively, to show a percentage of total time investigating each partner.

### Retrobead surgery

In a separate set of experiments, red retrobead IX (Lumafluor, Inc.; Durham, NC, United States) was infused into the septum or nucleus accumbens (NAc). Rats were first anesthetized with 4% isoflurane and oxygen until deeply anesthetized and maintained at 1.5–2.5% for the remainder of the surgery. A 10 μL Hamilton syringe with a 26-gauge needle (World Precision Instruments) containing the retrobeads was mounted onto a stereotaxic infusion pump (World Precision Instruments, Sarasota, FL, United States). All groups received bilateral infusions of retrobead (0.5 μL/side; 50 nanoliters/min) into the septum (adolescent: AP: −0.15, ML: 1.6; V: 6.6; adult: AP: −0.15, ML: 1.7, V: −6.6 at a 10° angle) or NAc (adolescent: AP: + 2.0, ML: 1.4; V: 6.7; adult: AP: + 2.0, ML: 1.6, V: −6.7) from bregma (Watson, 2007). The syringe was left in place for an additional 5 min following virus infusion to allow for diffusion. Approximately 1 week later, animals were isolated for 2 h and were then perfused for immunofluorescent quantification of the immediate early gene zif268 as a proxy of cellular activity.

### Immunofluorescence

Rats were deeply anesthetized following the brief isolation manipulation or control home cage and perfused with 0.1 M phosphate-buffered saline (PBS) followed by 4% paraformaldehyde. Brains were sliced on a vibratome in 40 μm sections and mounted onto gelatinized slides. Slides were rehydrated in wash buffer (PBS + 0.05% Tween-20), and endogenous peroxidase activity was blocked (PBS + 0.3% H_2_O_2_), and slices were permeabilized (PBS + 0.03% Triton X). Slices were then incubated in blocking solution for 1 h (PBS + 0.7% normal goat serum), and then EGR1 (zif268) antibody (Cell Signaling, 1:500, #15F7) overnight at 4°C. Slices were then incubated in a secondary solution (1:500, Alexa Fluor 488, Invitrogen, Waltham, MA, catalog #: A32731) for 2 h, rinsed with wash buffer, and coverslipped with a DAPI counterstain.

### Microscopy

Amygdala regions were identified based on the rat brain atlas (Watson, 2007). Basal, medial, and basomedial amygdala regions were captured on a Nikon Eclipse E600 microscope (Melville, NY, United States) using a 10× objective lens. Three square sections in each region were captured and analyzed bilaterally by an individual blind to condition. Images were then exported as TIFF files, and particles were quantified using ImageJ software (NIH, Bethesda, MD, United States). Zif268 and retrobead particles were counted by using the subtract background function and difference of Gaussian filtering and were then made binary. The watershed function was then used to separate overlapping particles, and particles were counted using the “Analyze Particles” function with a circularity between 0.04 and 1.00 (similar to Ferrara et al., 2019). Retrobeads were similarly quantified using ImageJ software particle counts with the exception that a Kuwahara filter was applied to reduce speckles with edges of retrobeads left intact. Retrobead and zif268 binary images were then added as Image A and Image B regions in the “Just Another Colocalization Plugin” in ImageJ, and objects-based method analyses were performed to identify the number of zif268-positive cells overlapping with retrobead particles. For each slice, zif268+ and retrobead numbers were normalized to DAPI. For each amygdala subregion, the zif+ to DAPI ratio in the no isolation homecage condition was averaged, and each subregion in isolation conditions was divided by the homecage average and multiplied by 100 to give a percent increase in zif268+ cells in isolated conditions from the no isolation.

### Statistical analyses

GraphPad Prism 8.0 (San Diego, CA, United States) was used for statistical analyses and figures. Data are represented as group averages with standard error of the mean (SEM). Behavioral experiments were analyzed using an unpaired *t*-test to compare social interaction between non-isolated and isolated groups, one-sample *t*-test to compare discrimination index values to chance investigation (e.g., 0), a two-way repeated measures ANOVA between conditions, and an F-test to compare the global fit of a second-order polynomial curve across time during social interaction. Correlations were analyzed with simple linear regression and a comparison of slopes from a line fitted to zero. For correlations, the percentage of time spent investigating was calculated by adding the time spent engaging in nose–body contact and anogenital sniffing and creating a percentage based on total behavior. Immunofluorescent data were analyzed using a one-way or two-way ANOVA. Fisher’s LSD *post hoc* analyses were used where main effects or interactions were observed.

## Results

### Facilitated social interaction contributes to increased discrimination between novel and familiar partners within briefly isolated adolescents

**FIGURE 1:**
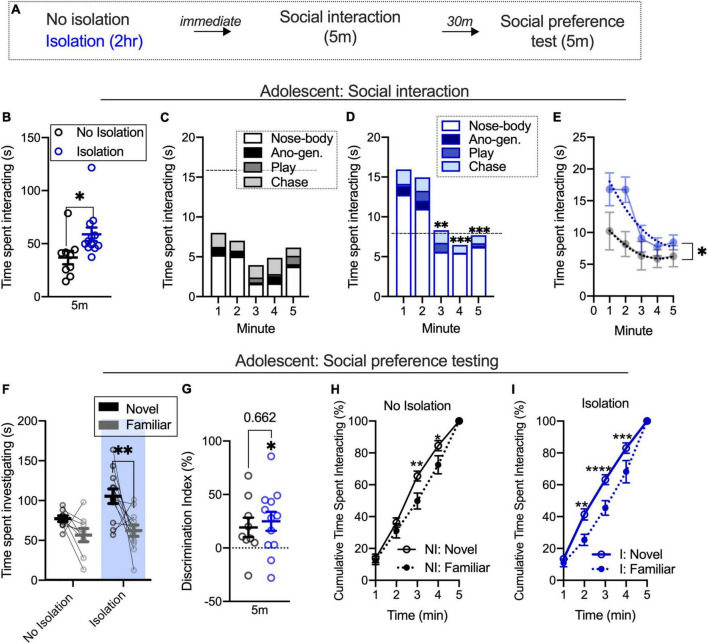
Facilitated social interaction contributes to increased discrimination between novel and familiar partners within briefly isolated adolescents. Schematic outlining both isolated (2 h) and non-isolated groups undergo social interaction and 30-min later social preference testing **(A)**. Social isolation increases social interaction **(B)**. Social interaction is similar across time in non-isolated adolescents **(C)**. Social interaction decreases in isolated adolescents over time **(D)**. Fitted lines significantly differ between isolated and non-isolated adolescents **(E)**. Isolation increases the time spent investigating a novel, relative to a familiar, partner during a novel partner test **(F)** also evidenced in a discrimination index **(G)**. Non-isolated adolescents show an elevated cumulative time spent investigating a novel partner relative to a familiar **(H)**, and isolated adolescents show a similar pattern consistent across time **(I)**. No isolation *n* = 9, isolation *n* = 12. *p < 0.05, **p < 0.01, ***p < 0.001, ****p < 0.0001.

We used a brief, 2-h isolation to determine the impact of changes in the social environment on social interaction and subsequent partner preference (Figure 1A). We found that isolated adolescents spent a greater amount of time socially interacting than their non-isolated counterparts [*t*_(19)_ = 2.384, *p* = 0.0277; No isolation *n* = 9, isolation *n* = 12; Figure 1B]. We next looked at the time spent investigating during each minute of the social interaction session. Using a repeated measures two-way ANOVA with time (5 m familiarization) and condition (no isolation, isolation) as factors, we found the main effect of time [*F*_(4,76)_ = 6.519, *p* = 0.0001] and condition [*F*_(1,19)_ = 5.682, *p* = 0.0277], but no interaction [*F*_(4,76)_ = 1.490, *p* = 0.214]. *Post hoc* analyses comparing the first minute of each condition to the remaining minutes found that isolated rats reduced social interaction from minute 1 to minute 3 (*p* = 0.0011) and to minute 4 (*p* = 0.0002) and to the final minute 5 (*p* = 0.0005; Figure 1D). There were no significant differences for minute-by-minute comparisons in the no isolation condition (Figure 1C). This difference in the pattern was confirmed by the examination of the distribution of interaction across time using a cumulative time histogram [*F*-test to compare the global fit of a second-order polynomial least squares line for isolation and no isolation conditions; *F*_(3,99)_ = 4.840, *p* = 0.0035; Figure 1E]. These results replicate prior work showing the facilitation of social interaction following 2-h isolation and extend this to demonstrate that different patterns of social behavior across time can occur among isolated and non-isolated rats, though these changes in interaction patterns are not dependent on the social environment as there was no significant interaction between condition and time.

We next used a two-way ANOVA with condition (no isolation, isolation) and target (novel partner, familiar partner) as factors to understand the impact of isolation on social partner preference. In adolescents, we found the main effect of condition [*F*_(1,19)_ = 11.69, *p* = 0.0029] and the main effect of target [*F*_(1,19)_ = 10.68, *p* = 0.004], but no interaction between the two [*F*_(1,19)_ = 1.361, *p* = 0.2579]. *Post hoc* analyses found that isolated adolescents spent more time investigating a novel partner relative to a familiar partner (*p* = 0.0031; Figure 1F). We also compared discrimination index values between conditions using an unpaired *t*-test and found no difference between groups [*t*_(19)_ = 0.444, *p* = 0.662; Figure 1F]. However, using a one-sample *t*-test, we found discrimination index [*t*_(11)_ = 2.818, *p* = 0.0167; Figure 1G] values differed when compared to equal levels of partner exploration, while non-isolated rats did not [*t*_(8)_ = 2.126, *p* = 0.0662] differ from this. Due to within-condition differences, we were next interested in understanding alterations in the distribution of time spent investigating novel and familiar partners within isolated and non-isolated groups, as shifts in this pattern over the course of time may help clarify the main effects of the condition and partner preference. Using a two-way repeated measures ANOVA with time and condition (no isolation, isolation) as factors, we found the main effect of time [*F*_(4,32)_ = 403.6, *p* < 0.001] but no main effect of condition [*F*_(1,8)_ = 3.942, *p* = 0.0823], and no interaction [*F*_(4,32)_ = 2.328, *p* = 0.0774]. *Post hoc* analyses found significant differences at minutes 3 (*p* = 0.0013) and 4 (*p* = 0.0108) in non-isolated adolescents, indicating differences in the rate at which a novel and a familiar partner is investigated at that time (Figure 1H). In the isolated group, there was a main effect of time [*F*_(4,44)_ = 390.8, *p* < 0.001], main effect of condition [*F*_(1,11)_ = 7.234, *p* = 0.021], and an interaction [*F*_(4,44)_ = 4.233, *p* = 0.0055]. *Post hoc* analyses found significant differences between novel and familiar partner cumulative time spent interacting during minutes 2 (*p* = 0.0003), 3 (*p* < 0.0001), and 4 (*p* = 0.0006; Figure 1I). These cumulative plots normalize for differences in time spent investigating novel and familiar partners, and illustrate that disproportionally more time is spent with novel partners after 2 min in control but earlier in isolated rats. This suggests that within the condition, elevated discrimination index values (compared to no preference) are linked to different distributions of preference across time even when total differences in time with each partner are accounted. Together with the social interaction training data, these results show that differences in the rate of social interaction over the course of time are accompanied by patterns of novel or familiar partner investigation within isolated and non-isolated rats quantified with cumulative novel and familiar interaction plots within each condition. However, without a significant interaction during social preference testing and only marginal impact using a discrimination index, brief isolation can influence the partner investigation but this condition does not necessarily influence the degree of social target investigation.

### Among briefly isolated adults, facilitated social interaction occurs alongside reductions in novel and familiar partner preference

**FIGURE 2:**
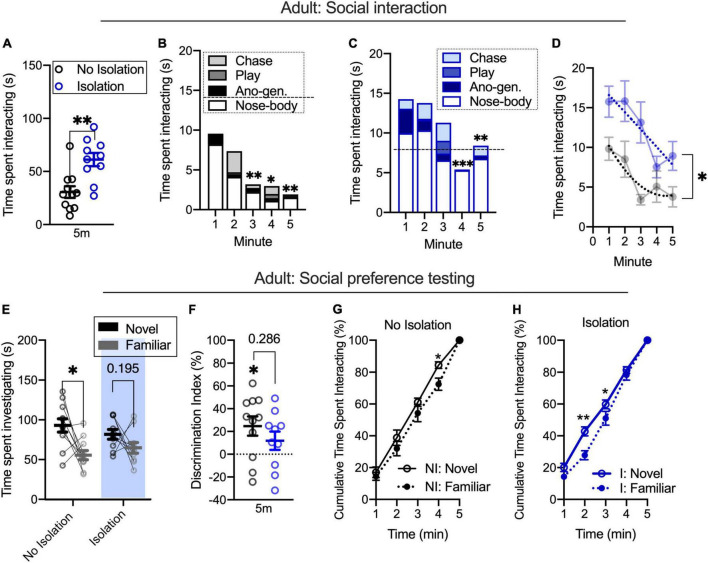
Among briefly isolated adults, facilitated social interaction occurs alongside shifted partner preference **(A)**. Social interaction decreases over time in non-isolated **(B)** and isolated **(C)** adults. Fitted lines significantly differ for the time spent interacting between conditions **(D)**. During partner preference testing, non-isolated adults spend more time investigating a novel relative to a familiar partner and this is reduced in the isolated condition **(E,F)**. Cumulative plots indicate different patterns of novel and familiar partner investigation across conditions, with isolated adults showing a greater difference in novel and familiar rates **(G,H)**. No isolation *n* = 11, isolation *n* = 10. *p < 0.05, **p < 0.01, ***p = 0.0007.

To understand the relationship in adults between social interaction during familiarization and social preference, the same design was tested in a different cohort of adult rats (as seen in Figure 1A). Replicating prior results, brief isolation increased the time spent socially interacting [*t*_(19)_ = 3.698, *p* = 0.0015; no isolation *n* = 11, isolation *n* = 10; Figure 2A]. We next used a two-way ANOVA with time (5-min familiarization) and condition (no isolation, isolation) as factors to understand differences in social interaction across time. We found the main effects of time [*F*_(4,76)_ = 7.497, *p* < 0.0001] and isolation [*F*_(4,76)_ = 13.67, *p* = 0.0015], but no interaction [*F*_(4,76)_ = 1.38, *p* = 0.2488]. *Post hoc* analyses showed that the non-isolated group show significantly lower social interaction in minutes 3 (*p* = 0.0052), 4 (*p* = 0.0356), and 5 (*p* = 0.0046) relative to the first minute (Figure 2B). In the isolated group, only the final 2 min were significantly different from the first minute (minute 4 *p* = 0.0007; minute 5 *p* = 0.0046; Figure 2C). An *F*-test for global fit was also significantly different between conditions [*F*_(4,76)_ = 9.329, *p* < 0.0001; Figure 2D] indicating differences in patterns of social interaction over the course of time within isolated and non-isolated conditions.

We next tested groups for novel or familiar partner investigation during a partner preference test. Using a repeated measures two-way ANOVA with condition (no isolation, isolation) and target (novel, familiar partner) as factors, we found the main effect of target [*F*_(1,19)_ = 9.739, *p* = 0.0056] but not condition [*F*_(1,19)_ = 0.0511, *p* = 0.8235] nor an interaction [*F*_(1,19)_ = 1.383, *p* = 0.2541]. *Post hoc* analyses found that the non-isolated group spent more time investigating a novel relative to a familiar partner (*p* = 0.0057) but did not in the isolated group (*p* = 0.1949; Figure 2E). Using a discrimination index to account for within-subjects changes in novel and familiar partner investigation, we did not find differences between conditions [*t*_(19)_ = 1.099, *p* = 0.286; Figure 2F], but we did find that non-isolated animals had significant discrimination index values [*t*_(10)_ = 2.936, *p* = 0.0149] compared to equivalent degree of novel and familiar investigation, whereas isolated groups did not [*t*_(9)_ = 1.479, *p* = 0.1734], indicating increased novelty preference in the non-isolated group that was not different between groups. To test differences in the distribution of time spent investigating a novel and familiar partner within the condition, we next used repeated measures two-way ANOVA with time (5-min partner preference test) and target (novel, familiar partner) as factors. In the non-isolated group, we found the main effect of time [*F*_(4,36)_ = 454.6, *p* < 0.0001] but no main effect of target [*F*_(1,9)_ = 2.576, *p* = 0.1429] nor an interaction [*F*_(4,36)_ = 0.8944, *p* = 0.4774]. *Post hoc* analyses revealed a significant difference at minute 4 (*p* = 0.0186). This result indicated that non-isolated rats showed a similar investigation preference across the session (Figure 2G). In the isolated group, we found the main effect of time [*F*_(4,40)_ = 792.6, *p* < 0.001] and an interaction [*F*_(4,40)_ = 2.647, *p* = 0.0473], but no main effect of target [*F*_(1,10)_ = 4.143, *p* = 0.0692]. *Post hoc* analyses found a significant difference between novel and familiar partners at minutes 2 (*p* = 0.0001) and 3 (*p* = 0.0179). This indicates that in isolated rats, the preference between novel and familiar partners shifts across the session (Figure 2H). Similar to adolescents, these results suggest that changes in the time spent socially interacting over the course of several minutes during a training period may be linked to distinct rates of novel and familiar partner investigation after isolation, even when investigation time is similar between partners, as indexed with cumulative plots. While the rate and degree of partner preference were different within conditions in adults, the degree of investigation between social targets is not dependent on the condition, as evidenced by a lack of interaction during social preference testing.

### Novel partner preference is related to social interaction and investigation in adults

**FIGURE 3:**
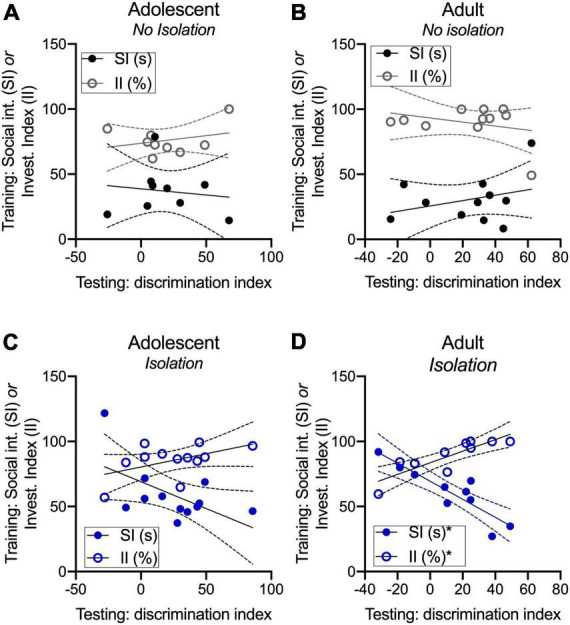
Novel partner preference is related to social interaction and investigation in adults. Social interaction and the percentage of time spent socially investigating are not related to discrimination index values in adult and adolescent non-isolated groups **(A,B)**. Time spent on social interaction was modestly correlated with discrimination index values, where a greater amount of social interaction was related to lower discrimination index values **(C)**. In isolated adults, discrimination index values were positively correlated with the investigation but negatively correlated with social interaction **(D)**. *p < 0.05.

We were next interested in whether social interaction, and specifically social investigation were associated with changes in novel partner preference measured with a discrimination index. We defined social interaction as the sum of all social behaviors over the course of the 5 m familiarization phase, and social investigation as the time spent engaging in nose–body contact and anogenital sniffing because odor cues form the basis of social recognition memory. In non-isolated groups, social interaction and the percentage of time spent investigating during familiarization were not correlated with partner discrimination at testing in adolescents [SI: R^2^: 0.01852, *F*_(1,7)_ = 0.1321, *p* = 0.727; II: R^2^: 0.082; *F*_(1,7)_ = 0.6253, *p* = 0.455; Figure 3A] and adults [SI: R^2^: 0.0965, *F*_(1,9)_ = 0.9616, *p* = 0.352; II: R^2^: 0.0881; *F*_(1,9)_ = 0.8693, *p* = 0.376; Figure 3B]. In isolated adolescents, the time spent socially interacting during the familiarization phase prior to testing was modestly correlated with partner discrimination at testing [R^2^: 0.3279; *F*_(1,10)_ = 4.878, *p* = 0.0517] but the percentage of time spent investigating was not [R^2^: 0.2418; *F*_(1,10)_ = 3.190, *p* = 0.1044; Figure 3C]. In isolated adults, social interaction during the familiarization phase prior to testing was negatively correlated [R^2^: 0.7931; *F*_(1,8)_ = 30.67, *p* = 0.0005] and the percentage of time spent investigating (nose–body and anogenital sniffing during familiarization) was positively correlated with partner discrimination at test [R^2^: 0.7269; *F*_(1,8)_ = 21.29, *p* = 0.0017; Figure 3D]. These results suggest that within-group changes among isolated adults and adolescents may be related to an emerging pattern where social interaction itself is inversely but the investigation is positively correlated with novel partner preference.

### Isolation increases activity in adolescent basal amygdala-nucleus accumbens and -septal circuits relative to the BMA and MeA amygdala

**FIGURE 4:**
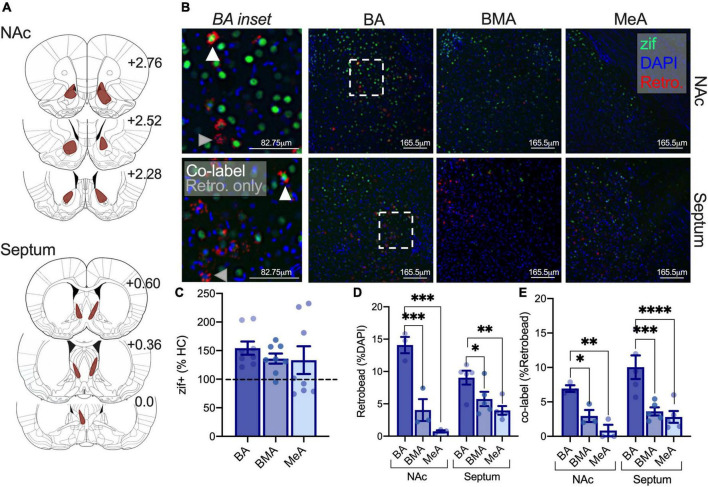
Isolation increases activity in adolescent BA-NAc and -septal circuits relative to the BMA and MeA amygdala. Reconstruction of retrobead injections in the NAc and septum of adolescents **(A)**. Representative images depicting zif268 expression in green, retrobead expression in red, and DAPI in blue in the BA, BMA, and MeA. BA insets show co-labeled zif268+ retrobead cells at the tip of white triangles and retrobead only cells at the tip of gray triangles **(B)**. Zif268 expression is not significantly different in the BA, BMA, or MeA of isolated adolescents **(C)**. Retrobead density is highest in the BA for septum and NAc projections **(D)**. Isolation increases the number of BA-NAc and BA-septum zif268 expressing cells relative to the BMA **(E)**. Homecage *n* = 3, isolated NAc retrobead *n* = 3, isolated septum retrobead *n* = 5. *p < 0.05, **p < 0.01, ***p < 0.001, ****p < 0.0001.

Our prior work shows that isolation increases activity in lateral and basal regions of the amygdala, and others have demonstrated critical roles for the NAc and septum in social behavior (Everts and Koolhaas, 1999; Manduca et al., 2016; Ferrara et al., 2021b). We were next interested in whether isolation differentially engages the activity of amygdala-NAc and -septal circuits sensitive to social circumstances to understand the impact of isolation on partner preference. Adult and adolescent rats were infused with a retrobead into the NAc or the septum and were isolated to quantify changes in (1) amygdala subregions engaged by isolation using immediate early gene zif268 as a proxy for cellular activity, (2) density of neurons projecting to the NAc and septum through retrobead quantification, and (3) the proportion of cells activated and projecting to the NAc or septum as a result of isolation (Figures 4A,B). In adolescents, a repeated measures one-way ANOVA to assess isolation-driven increases in zif268 expression in the BA, BMA, and MeA subregions of the amygdala found no differences in effects of isolation across amygdala subregions [*F*_(2,14)_ = 0.829, *p* = 0.457; NAc retrobead injection *n* = 3, septum retrobead injection *n* = 5; Figure 4C] nor when comparisons included homecage conditions [highest *F*: main effect of condition *F*_(1,9)_ = 1.583, *p* = 0.2400]. These results indicate that there is a more marginal and variable impact of isolation on immediate early gene activity across amygdala subregions in adolescents. A repeated measures two-way ANOVA was used with retrobead injection site (NAc, Septum) and amygdala subregion (BA, BMA, MeA) as factors to test differences in the density of amygdala projecting NAc and septum neurons in adolescents. We found a significant main effect of amygdala subregion [*F*_(2,12)_ = 37.49, *p* < 0.0001] and an interaction [*F*_(2,12)_ = 8.055, *p* = 0.0061], but no main effect of retrobead injection site [*F*_(1,6)_ = 0.0009, *p* = 0.9776]. *Post hoc* comparisons show a greater number of BA neurons projecting to the NAc relative to BMA (*p* < 0.0001) and MeA (*p* < 0.0001), as well as significantly more BA neurons projecting to the septum relative to BMA (*p* = 0.036) and MeA (*p* = 0.0306; Figure 4D). To understand whether isolation differentially engages adolescent amygdala-septal and -NAc circuits, we next quantified the number of cells labeled with both zif268 and a retrobead. Using a repeated measures two-way ANOVA, we found a significant main effect of amygdala subregion [*F*_(2,12)_ = 25.51, *p* < 0.001] but no main effect of retrobead site [*F*_(1,6)_ = 2.577, *p* = 0.1595] nor an interaction [*F*_(2,12)_ = 0.7784, *p* = 0.481]. *Post hoc* comparisons found a greater percent of zif268 co-labeled with BA neurons projecting to the NAc relative to BMA (*p* = 0.0248) and MeA (*p* = 0.002), and a greater percent of zif268 co-labeled with BA neurons projecting to the septum relative to BMA (*p* = 0.0002) and to the MeA (*p* < 0.0001; Figure 4E). These results indicate that there are similar patterns of activity across amygdala subregions, and BA-NAc and -septum circuits may be preferentially engaged relative to the BMA and MeA to support isolation-driven changes in social interaction and partner preference.

### Isolation selectively increases adult basal amygdala activity but does not preferentially engage nucleus accumbens or septal amygdala circuits

**FIGURE 5:**
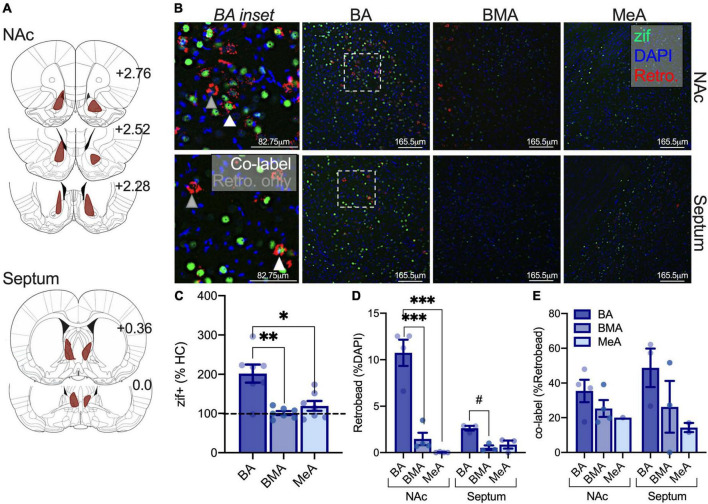
Isolation selectively increases adult BA activity but does not preferentially engage NAc or septal amygdala circuits. Retrobead expression reconstructions for NAc and septum injections in adults **(A)**. Representative immunofluorescent images of the BA, BMA, and MeA with zif268 (green), retrobeads from the NAc or septum (red), and DAPI (blue). BA insets indicate retrobead only neurons at the tip of gray triangles, and zif+ retrobead co-labeled cells at the tip of white triangles **(B)**. Isolation increases zif268 expression in the BA relative to the BMA and MeA of adults **(C)**. Retrobead density is highest in BA relative to the BMA and MeA when retrobeads were infused into the NAc, and retrobead density is only modestly higher in the BA relative to the BMA when infused into the septum **(D)**. There were no significant differences in the activity of amygdala-NAc or -septum circuits following isolation in adults **(E)**. Homecage *n* = 3, isolated NAc retrobead *n* = 4, isolated septum retrobead *n* = 3. #*p* = 0.081, *p < 0.05, **p < 0.01, ***p < 0.0001.

We next investigated differences in the sensitivity of amygdala-NAc and -septum neurons to isolation using the same approach as above (Figures 5A,B). When normalized to homecage averages, a one-way repeated measures ANOVA found differences in zif268 expression between amygdala subregions as a result of isolation in adults [*F*_(2,12)_ = 12.87, *p* = 0.0038; NAc retrobead injection *n* = 4, septum retrobead injection *n* = 3]. *Post hoc* comparisons found that BA zif268 expression was elevated relative to the BMA (*p* = 0.0034) and MeA (*p* = 0.0192; Figure 5C). When including comparisons with homecage groups, we found the main effect of condition [*F*_(1,8)_ = 6.400, *p* = 0.0353], amygdala subregion [*F*_(2,16)_ = 4.867, *p* = 0.0223], and an interaction [*F*_(2,16)_ = 4.867, *p* = 0.0223] with a significant increase in BA activity after isolation relative to homecage (*p* = 0.0006). We next used a repeated measures two-way ANOVA to investigate differences in the density of amygdala neurons projecting to the NAc and septum with amygdala subregion (BA, BMA, and MeA) and retrobead injection site (NAc, septum) as factors. There was a significant main effect of amygdala subregion [*F*_(2,10)_ = 46.72, *p* < 0.0001], main effect of retrobead site [*F*_(1,5)_ = 14.26, *p* = 0.0129], and an interaction between the two [*F*_(2,10)_ = 22.03, *p* = 0.0002]. Similar to adolescents, follow-up comparisons found a greater density of BA neurons projecting to the NAc relative to BMA (*p* < 0.0001) and MeA (*p* < 0.0001), but only modestly more BA-septum neurons relative to BMA (*p* = 0.081; Figure 5D). We next used a mixed model two-way ANOVA with the same factors (instead of repeated measures, due to uneven within subjects matching because retrobeads were not detected across all amygdala subregions in all subjects). Using this two-way ANOVA, there was no main effect of retrobead injection site [*F*_(1,5)_ = 0.2199, *p* = 0.6588], no main effect of amygdala subregion [*F*_(1.867,5.601)_ = 4.884, *p* = 0.0551], and no interaction [*F*_(2,6)_ = 0.5501, *p* = 0.6034; Figure 5E]. These results suggest that isolation preferentially engages the adult BA relative to other amygdala subregions, with a similar degree of activation in amygdala-NAc and -septal following isolation.

## Discussion

Our current results outline the impact of brief isolation on partner preference and amygdala circuits sensitive to isolation. Though the degree of novel partner preference did not depend on isolation conditions, we did observe a number of within-group alterations that shed light on how social interaction is related to partner preference in adults and adolescents. Specifically, we found that novel partner preference within isolated groups was related to the duration of social investigation but was inversely related to global social interaction, and this effect increases with age. These results suggest that isolation itself does not impact partner preference; rather there are within-cohort differences in partner preference that are related to social interaction behaviors. We also identified two distinct amygdala circuits sensitive to isolation that may contribute to alterations in social interaction and preference within isolated and non-isolated conditions. In isolated adolescents, there were no differences in activity (measured with immediate early gene, zif268, expression) in the BA, BMA, and MeA, but the activity of BA neurons projecting to the septum and the NAc relative to MeA and BMA regions were increased under isolation conditions. In adults, isolation selectively increases activity in the BA relative to the BMA and MeA and does not preferentially impact amygdala BA, BMA, or MeA neurons projecting to the NAc or septum. These results suggest that when isolated, the degree of activity between regions sensitive to social circumstances and circuits within these regions are differentially impacted with age that may influence social behavior, where social behaviors facilitated by isolation contribute to an emerging relationship with partner preference selectively within isolated conditions.

Within isolated and non-isolated groups, we found that isolated adolescents show a reduction in time spent interacting over time and this effect is absent in non-isolated adolescents, though this change over time is not dependent on isolation. In adults, we identify a reduction in social interaction over the course of time within both non-isolated and isolated groups, but within condition, comparisons did show that non-isolated adults show a longer duration of this reduction. Within (but not between) these groups, we also found differences in social preference patterns over time, linked to the degree of novel and familiar partner investigation at later times when compared to equivalent novel and familiar investigation levels. The larger reductions in social interaction over time in non-isolated adults and isolated adolescents provide evidence for within-group dynamics that are related to subsequent discrimination, as these two groups show improved novel partner preference. Shifts in the degree of interaction as a result of stress influence social preference (Hodges et al., 2017; Eacret et al., 2019). Our results add to this literature and suggest that a degree of habituation or familiarization within (but not between) a group, indexed with a persistent reduction in interaction from initial interaction levels, to a partner may improve discrimination between novel and familiar partners.

Isolation not only influences the degree of interaction but also increases the number of social behaviors across ages that are known to hold rewarding properties (e.g., social play) and may shift partner preferences toward familiar partners. The increase in a host of behaviors in isolation-driven states, particularly between ages that show dramatic changes in social behavior, provides behavioral mechanisms for social preference. In line with this, isolated and non-isolated adolescents show socially conditioned place preference, whereas adults only show socially conditioned place preference following isolation, indicating group differences in the rewarding nature of social interactions (Douglas et al., 2004). It has also been suggested that there are specific aspects of social interaction aside from social play that can drive social preference and reward, linking all adolescent social interaction, not limited to play, to social preference (Thiel et al., 2008; Peartree et al., 2012). Further, individual differences in fighting and chase versus investigation-like behaviors contribute to dynamic social hierarchies, highlighting the importance of individual differences and perhaps a balance in these behaviors that may promote shifts in social preference (So et al., 2015). In the current study, we found that the percentage of time spent socially investigating is positively related to novel partner preference, while total social interaction (e.g., play, chase, investigation) is inversely correlated with partner preference in isolated adults. Interestingly, this pattern is relatively weak in adolescents and becomes stronger with social maturation, indicating developmental differences and alterations in the social repertoire over time that are related to novel partner preference in line with the idea that the nature and value of social interactions change over the course of development.

Neural circuits sensitive to isolation form a network that changes with isolation, and it has been proposed that isolation shifts the activation of this larger neural circuit to preserve/maintain social homeostasis (Lee et al., 2021). We previously identified the basolateral amygdala as a critical region regulating isolation-driven changes in social behavior across ages (Ferrara et al., 2021b), and several other amygdala subregions and outputs have been implicated in social behavior and sensitivity to the social environment, with relatively little known about changes in activity during development (Everts and Koolhaas, 1999; Manduca et al., 2016; Mesquita et al., 2016; Twining et al., 2017). To understand whether a broader amygdala network may be sensitive to the isolation that subsequently impacts adults and adolescents differently, we first quantified activity in the BA, BMA, and MeA following brief isolation. We found that adolescents show similar levels of activity between amygdala subregions following isolation, and adults show a selective increase in BA activity relative to the BMA and MeA. Within the BA, NAc- and septum-projecting neurons show elevated activity following isolation in adolescents relative to the BMA and MeA but no such differences were seen in adults. Interestingly, BA-NAc and -septum adult activation are higher than adolescent activation. This may indicate that both -NAc and -septum projections are sensitive to isolation in adults or alternatively that other amygdala projections may show a more selective subregion activation, as seen in our adolescent cohort. Future work should investigate the contribution of these amygdala projections or alternative projections to isolation-driven changes in social behavior. These results suggest that adolescents may be more sensitive to changes in the social environment, observed with heightened activity in BA-NAc and BA-septal circuits relative to other amygdala subregions, which may contribute to partner preference following brief isolation in adolescents. In line with this, activity in a BA-septum circuit is required for novel partner preference, while increases in BA-NAc circuits may govern the rewarding nature of social interactions (Amaral et al., 2021; Rodriguez et al., 2021). In adults, overall similar levels of amygdala-NAc and -septum activity may contribute to the persistent levels of social interaction over time and subsequent reductions in novel partner preference relative to a familiar partner within, but not between, isolated and non-isolated groups. However, we note that exposure to a novel environment itself can drive increases in immediate early gene expression, which may contribute to isolation-driven changes in zif268 protein observed here. This increase has been demonstrated in the amygdala for novel context encoding, as well as for context fear memory, and the amygdala can regulate immediate early gene expression in other brain regions following context learning (Rosen et al., 1998; Huff et al., 2006; Asok et al., 2013; Antoine et al., 2014; Heroux et al., 2018). Based on this, it is possible that the increased zif268 activity observed here is produced by a change in the environment rather than by social deprivation. However, studies probing for zif268 protein (as in the present study), rather than mRNA [as in Asok et al. (2013) and Heroux et al. (2018)], found no such increase in zif268 expression (e.g., Trask and Helmstetter, 2022), suggesting that while this is possible in the present manuscript, novel context exploration is likely not the sole driver of zif268 activity.

Together, we provide evidence that isolation uniformly increases social interaction and differentially alters patterns of amygdala activity within isolated adults and adolescents. However, non-isolated and isolated cohorts show different patterns of social interaction and activation of BA-NAc and -septum circuits that may contribute to shifts in social preference within isolated adults and adolescents. We provide a framework for behavioral and biological mechanisms that are differentially impacted by age that ultimately sheds light on the importance of the social environment for social memory.

## Data availability statement

The original contributions presented in this study are included in the article/supplementary material, further inquiries can be directed to the corresponding author.

## Ethics statement

The animal study was reviewed and approved by Institutional Animal Care and Use Committee at Rosalind Franklin University of Medicine and Science.

## Author contributions

NF and JR: conceptualization and methodology. NF, ST, AR, and MP: data curation. NF: wrote the original draft and formally analyzed the data. NF, ST, AR, and JR: reviewed and edited the manuscript. All authors contributed to the article and approved the submitted version.
